# *catena*-Poly[tris­(μ_2_-1*H*-benzimidazole-5-carboxyl­ato)europium(III)]

**DOI:** 10.1107/S1600536810051706

**Published:** 2010-12-15

**Authors:** Junlin Gao, Jun Wang, Chuntao Dai

**Affiliations:** a Zhongshan Polytechnic, Zhongshan, Guangdong 528404, People’s Republic of China

## Abstract

In the title one-dimensional coordination polymer, [Eu(C_8_H_5_N_2_O_2_)_3_]_*n*_, the Eu^III^ ion is eight-coordinated by the carboxyl­ate O atoms of six ligands in a distorted monocapped penta­gonal–bipyramidal geometry. The ligands link Eu^III^ ions, forming helical chains parallel to the *c* axis, with Eu⋯Eu separations of 4.0678 (11) Å. The chains are further inter­connected by N—H⋯N hydrogen bonds into a three-dimensional supra­molecular network. The crystal studied was a racemic twin, as suggested by the Flack parameter of 0.367 (14).

## Related literature

For general background to coordination polymers based on *N*-heterocyclic carboxyl­ates, see: Huang *et al.* (2009 ▶); Cheng *et al.* (2008 ▶); Qiu *et al.* (2007 ▶). For related structures, see: Guo, Cao *et al.* (2007 ▶); Guo, Li *et al.* (2007 ▶); Peng *et al.* (2010 ▶).
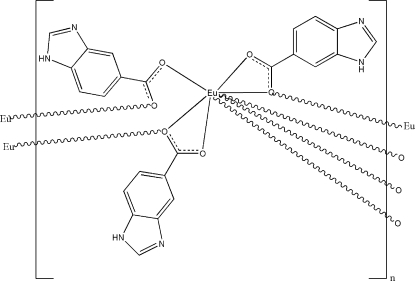


## Experimental

### 

#### Crystal data

[Eu(C_8_H_5_N_2_O_2_)_3_]
*M* *_r_* = 635.38
Monoclinic, 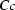

*a* = 23.211 (2) Å
*b* = 12.4312 (12) Å
*c* = 8.1329 (8) Å
β = 107.902 (1)°
*V* = 2233.1 (4) Å^3^
*Z* = 4
Mo *K*α radiation
μ = 2.87 mm^−1^
*T* = 293 K
0.20 × 0.19 × 0.13 mm

#### Data collection

Bruker APEXII area-detector diffractometer
Absorption correction: multi-scan (*SADABS*; Sheldrick, 2008 ▶) *T*_min_ = 0.598, *T*_max_ = 0.707
5623 measured reflections
3595 independent reflections
3412 reflections with *I* > 2σ(*I*)
*R*_int_ = 0.019

#### Refinement

*R*[*F* ^2^ > 2σ(*F* ^2^)] = 0.022
*wR*(*F* ^2^) = 0.054
*S* = 1.05
3595 reflections
335 parameters
2 restraints
H-atom parameters constrained
Δρ_max_ = 1.07 e Å^−3^
Δρ_min_ = −0.66 e Å^−3^
Absolute structure: Flack (1983 ▶), 1587 Friedel pairs
Flack parameter: 0.367 (14)

### 

Data collection: *APEX2* (Bruker, 2004 ▶); cell refinement: *SAINT* (Bruker, 2004 ▶); data reduction: *SAINT*; program(s) used to solve structure: *SHELXS97* (Sheldrick, 2008 ▶); program(s) used to refine structure: *SHELXL97* (Sheldrick, 2008 ▶); molecular graphics: *SHELXTL* (Sheldrick, 2008 ▶); software used to prepare material for publication: *SHELXTL*.

## Supplementary Material

Crystal structure: contains datablocks I, global. DOI: 10.1107/S1600536810051706/rz2533sup1.cif

Structure factors: contains datablocks I. DOI: 10.1107/S1600536810051706/rz2533Isup2.hkl

Additional supplementary materials:  crystallographic information; 3D view; checkCIF report

## Figures and Tables

**Table 1:** Hydrogen-bond geometry (Å, °)

*D*—H⋯*A*	*D*—H	H⋯*A*	*D*⋯*A*	*D*—H⋯*A*
N3—H3*A*⋯N2^i^	0.86	1.96	2.798 (6)	165
N5—H5⋯N4^ii^	0.86	2.04	2.839 (6)	155
N1—H1⋯N6^ii^	0.86	1.97	2.827 (6)	173

Symmetry codes: (i) 
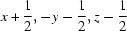
; (ii) 
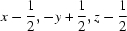
.

**Fig. 1.**
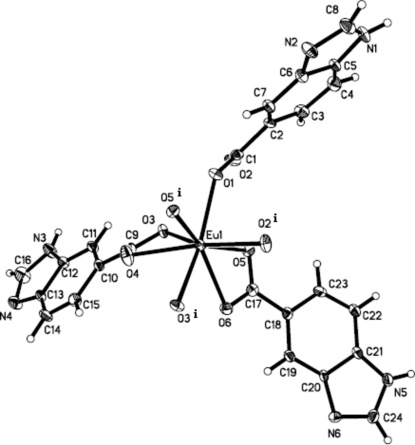
The asymmetric unit of the title compound, showing 30% probability displacement ellipsoids. Symmetry code: (i) x, -y, 0.5+z.

**Fig. 2.**
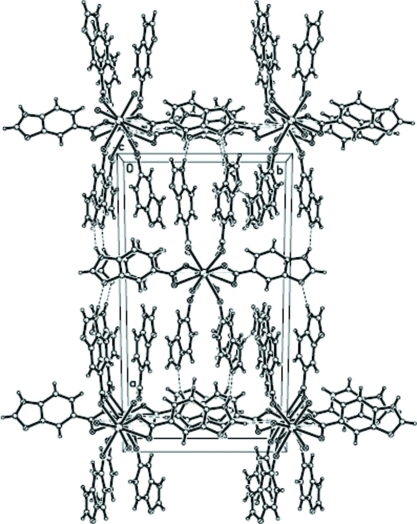
Crystal packing of the title compound viewed along the c axis. Intermolecular hydrogen bonds are shown as dashed lines.

## supplementary crystallographic information

### Comment

Metal coordination polymers based on N-heterocyclic carboxylates such as pyridinecarboxylates have raised intense interest for their structural diversity and their potential applications as functional materials (Huang *et al.*, 2009; Cheng *et al.*, 2008; Qiu *et al.*, 2007). To date, one-, two-, and three-dimensional coordination polymers have been synthesized by the choice of appropriate metal ions and versatile benzimidazole carboxylates as ligands (Guo, Cao *et al.*, 2007; Guo, Li *et al.*, 2007b; Peng *et al.*, 2010). Herein, the hydrothermal treatment of Eu_2_O_3_ with 1*H*-benzimidazole-5-carboxylic acid led to a the synthesis of the novel title compound, [Eu(C_8_H_5_N_2_O_2_)_3_]_n_, whose structure is reported herein.

As depicted in Fig. 1, the europium(III) ion is eight-coordinated by the O atoms of six 1*H*-benzimidazole-5-carboxylate ligands, adopting a distorted monocapped pentagonal-bipyramidal geometry. The 1*H*-benzimidazole-5-carboxylate ligands link europium ions, forming helical chains parallel to the *c* axis. The Eu···Eu separations within the polymer are 4.0678 (11) /%A. These chains are further interconnected by N—H···N hydrogen bonds and extend to form a three-dimensional supramolecular network (Table 1; Fig. 2).

### Experimental

A mixture of Eu_2_O_3_ (0.070 g, 0.2 mmol), 1*H*-benzimidazole-5-carboxylic acid (0.193 g, 1.2 mmol), and H_2_O (10 mL) was sealed in a 20 mL Teflon-lined reactor, which was heated in an oven to 423 K for 48 h and then cooled to room temperature at a rate of 5 K h^-1^. Colourless crystals suitable for X-ray analysis were obtained in a yield of 76% based on Eu.

### Refinement

The crystal studied was a racemic twin, as suggested by the Flack parameter of 0.367 (14) obtained by the TWIN/BASF procedure in SHELXL (Sheldrick, 2008). All H atoms were fixed geometrically and treated as riding, with C—H = 0.93 Å, N—H = 0.86 Å, and with U_iso_(H) = 1.2U_eq_(C, N).

### Crystal data

**Table:**

[Eu(C_8_H_5_N_2_O_2_)_3_]	*F*(000) = 1248
*M**_r_* = 635.38	*D*_x_ = 1.890 Mg m^−^^3^
Monoclinic, *C**c*	Mo *K*α radiation, λ = 0.71073 Å
Hall symbol: C -2yc	Cell parameters from 2300 reflections
*a* = 23.211 (2) Å	θ = 1.2–28.0°
*b* = 12.4312 (12) Å	µ = 2.87 mm^−^^1^
*c* = 8.1329 (8) Å	*T* = 293 K
β = 107.902 (1)°	Block, colourless
*V* = 2233.1 (4) Å^3^	0.20 × 0.19 × 0.13 mm
*Z* = 4	

### Data collection

**Table:**

Bruker APEXII area-detector diffractometer	3595 independent reflections
Radiation source: fine-focus sealed tube	3412 reflections with *I* > 2σ(*I*)
graphite	*R*_int_ = 0.019
φ and ω scans	θ_max_ = 25.2°, θ_min_ = 1.8°
Absorption correction: multi-scan (*SADABS*; Sheldrick, 2008)	*h* = −27→27
*T*_min_ = 0.598, *T*_max_ = 0.707	*k* = −12→14
5623 measured reflections	*l* = −9→9

### Refinement

**Table:**

Refinement on *F*^2^	Secondary atom site location: difference Fourier map
Least-squares matrix: full	Hydrogen site location: inferred from neighbouring sites
*R*[*F*^2^ > 2σ(*F*^2^)] = 0.022	H-atom parameters constrained
*wR*(*F*^2^) = 0.054	*w* = 1/[σ^2^(*F*_o_^2^) + (0.0245*P*)^2^ + 1.858*P*] where *P* = (*F*_o_^2^ + 2*F*_c_^2^)/3
*S* = 1.05	(Δ/σ)_max_ < 0.001
3595 reflections	Δρ_max_ = 1.07 e Å^−^^3^
335 parameters	Δρ_min_ = −0.66 e Å^−^^3^
2 restraints	Absolute structure: Flack (1983), 1587 Friedel pairs
Primary atom site location: structure-invariant direct methods	Flack parameter: 0.367 (14)

### Special details

**Table:**

Geometry. All esds (except the esd in the dihedral angle between two l.s. planes) are estimated using the full covariance matrix. The cell esds are taken into account individually in the estimation of esds in distances, angles and torsion angles; correlations between esds in cell parameters are only used when they are defined by crystal symmetry. An approximate (isotropic) treatment of cell esds is used for estimating esds involving l.s. planes.
Refinement. Refinement of F^2^ against ALL reflections. The weighted R-factor wR and goodness of fit S are based on F^2^, conventional R-factors R are based on F, with F set to zero for negative F^2^. The threshold expression of F^2^ > 2sigma(F^2^) is used only for calculating R-factors(gt) etc. and is not relevant to the choice of reflections for refinement. R-factors based on F^2^ are statistically about twice as large as those based on F, and R- factors based on ALL data will be even larger.

### Fractional atomic coordinates and isotropic or equivalent isotropic displacement parameters (Å^2^)

**Table:**

	*x*	*y*	*z*	*U*_iso_*/*U*_eq_	
C1	0.7810 (2)	−0.1030 (3)	0.3121 (6)	0.0246 (10)	
C2	0.7133 (2)	−0.1136 (3)	0.2747 (6)	0.0247 (10)	
C3	0.6748 (2)	−0.0808 (4)	0.1122 (7)	0.0320 (11)	
H3	0.6914	−0.0568	0.0285	0.038*	
C4	0.6124 (2)	−0.0839 (4)	0.0754 (7)	0.0350 (12)	
H4	0.5869	−0.0602	−0.0305	0.042*	
C5	0.58948 (19)	−0.1233 (4)	0.2012 (6)	0.0262 (10)	
C6	0.6284 (2)	−0.1580 (4)	0.3601 (6)	0.0266 (10)	
C7	0.69065 (19)	−0.1513 (4)	0.3995 (6)	0.0260 (10)	
H7	0.7161	−0.1717	0.5074	0.031*	
C8	0.5362 (2)	−0.1815 (4)	0.3580 (7)	0.0379 (12)	
H8	0.5029	−0.2007	0.3920	0.045*	
C9	0.9849 (2)	−0.0888 (3)	0.5739 (6)	0.0250 (10)	
C10	1.0465 (2)	−0.1076 (3)	0.5592 (6)	0.0235 (10)	
C11	1.0547 (2)	−0.1516 (4)	0.4114 (6)	0.0268 (10)	
H11	1.0219	−0.1689	0.3161	0.032*	
C12	1.1136 (2)	−0.1687 (4)	0.4115 (6)	0.0266 (10)	
C13	1.16339 (19)	−0.1397 (4)	0.5542 (6)	0.0272 (10)	
C14	1.1553 (2)	−0.0948 (4)	0.7007 (7)	0.0355 (12)	
H14	1.1882	−0.0749	0.7940	0.043*	
C15	1.0968 (2)	−0.0804 (4)	0.7041 (6)	0.0333 (12)	
H15	1.0902	−0.0525	0.8027	0.040*	
C16	1.1963 (2)	−0.2119 (5)	0.3592 (8)	0.0470 (15)	
H16	1.2226	−0.2391	0.3033	0.056*	
C17	0.88812 (19)	0.1921 (3)	0.4501 (6)	0.0238 (11)	
C18	0.8756 (2)	0.3027 (4)	0.3823 (7)	0.0235 (12)	
C19	0.9090 (2)	0.3881 (4)	0.4713 (7)	0.0248 (11)	
H19	0.9418	0.3766	0.5697	0.030*	
C20	0.8928 (5)	0.4911 (4)	0.411 (2)	0.0215 (18)	
C21	0.8393 (2)	0.5091 (3)	0.2693 (7)	0.0246 (11)	
C22	0.8063 (2)	0.4221 (4)	0.1781 (6)	0.0289 (11)	
H22	0.7722	0.4332	0.0834	0.035*	
C23	0.8256 (2)	0.3205 (4)	0.2321 (6)	0.0285 (11)	
H23	0.8056	0.2618	0.1690	0.034*	
C24	0.8778 (3)	0.6620 (4)	0.3649 (7)	0.0335 (16)	
H24	0.8834	0.7360	0.3760	0.040*	
Eu1	0.88497 (4)	0.004139 (14)	0.65258 (9)	0.01831 (7)	
N1	0.53063 (17)	−0.1384 (3)	0.2059 (6)	0.0357 (10)	
H1	0.4976	−0.1229	0.1264	0.043*	
N2	0.59260 (17)	−0.1958 (4)	0.4594 (6)	0.0347 (10)	
N3	1.13677 (18)	−0.2146 (4)	0.2901 (6)	0.0388 (10)	
H3A	1.1166	−0.2396	0.1904	0.047*	
N4	1.21553 (18)	−0.1670 (3)	0.5153 (6)	0.0372 (10)	
N5	0.83136 (19)	0.6180 (3)	0.2483 (6)	0.0307 (10)	
H5	0.8021	0.6508	0.1742	0.037*	
N6	0.91641 (18)	0.5911 (3)	0.4667 (6)	0.0296 (9)	
O1	0.81272 (13)	−0.1030 (3)	0.4680 (4)	0.0293 (7)	
O2	0.80206 (14)	−0.0948 (3)	0.1870 (4)	0.0340 (8)	
O3	0.93970 (13)	−0.0700 (3)	0.4411 (4)	0.0257 (7)	
O4	0.97779 (16)	−0.0890 (3)	0.7212 (4)	0.0416 (9)	
O5	0.86050 (14)	0.1111 (2)	0.3630 (4)	0.0240 (7)	
O6	0.92350 (15)	0.1775 (2)	0.6017 (4)	0.0302 (7)	

### Atomic displacement parameters (Å^2^)

**Table:**

	*U*^11^	*U*^22^	*U*^33^	*U*^12^	*U*^13^	*U*^23^
C1	0.025 (2)	0.022 (2)	0.030 (3)	−0.0051 (18)	0.013 (2)	−0.0025 (18)
C2	0.017 (2)	0.024 (2)	0.031 (3)	−0.0055 (17)	0.005 (2)	−0.0043 (18)
C3	0.032 (3)	0.036 (3)	0.029 (3)	−0.006 (2)	0.012 (2)	0.007 (2)
C4	0.034 (3)	0.040 (3)	0.027 (3)	0.002 (2)	0.003 (2)	0.007 (2)
C5	0.016 (2)	0.031 (2)	0.027 (3)	0.0007 (19)	0.001 (2)	−0.0004 (19)
C6	0.021 (2)	0.033 (3)	0.027 (3)	0.0007 (19)	0.008 (2)	0.000 (2)
C7	0.018 (2)	0.033 (3)	0.025 (3)	−0.0010 (18)	0.004 (2)	0.0056 (19)
C8	0.023 (3)	0.049 (3)	0.044 (3)	−0.003 (2)	0.016 (2)	−0.007 (3)
C9	0.024 (2)	0.025 (2)	0.024 (3)	0.0073 (19)	0.004 (2)	0.0018 (19)
C10	0.017 (2)	0.026 (2)	0.027 (3)	0.0025 (18)	0.005 (2)	−0.0010 (18)
C11	0.017 (2)	0.032 (2)	0.030 (3)	0.0000 (19)	0.005 (2)	−0.003 (2)
C12	0.024 (2)	0.030 (2)	0.025 (3)	−0.0008 (19)	0.006 (2)	−0.0020 (19)
C13	0.014 (2)	0.029 (2)	0.037 (3)	0.0010 (18)	0.005 (2)	0.000 (2)
C14	0.022 (3)	0.041 (3)	0.036 (3)	−0.001 (2)	−0.002 (2)	−0.013 (2)
C15	0.033 (3)	0.035 (3)	0.028 (3)	0.007 (2)	0.004 (2)	−0.011 (2)
C16	0.033 (3)	0.065 (4)	0.053 (4)	0.003 (3)	0.027 (3)	−0.007 (3)
C17	0.021 (2)	0.028 (2)	0.029 (3)	0.0003 (19)	0.018 (2)	−0.0008 (19)
C18	0.019 (3)	0.026 (2)	0.029 (3)	0.002 (2)	0.012 (3)	0.001 (2)
C19	0.018 (3)	0.028 (3)	0.028 (3)	0.000 (2)	0.005 (2)	0.001 (2)
C20	0.014 (5)	0.022 (2)	0.028 (3)	0.003 (2)	0.007 (4)	0.007 (2)
C21	0.016 (3)	0.030 (3)	0.026 (3)	0.0005 (17)	0.005 (2)	0.0037 (18)
C22	0.021 (2)	0.036 (3)	0.026 (3)	0.001 (2)	0.001 (2)	0.004 (2)
C23	0.022 (2)	0.034 (3)	0.028 (3)	−0.006 (2)	0.005 (2)	−0.006 (2)
C24	0.030 (4)	0.024 (3)	0.048 (5)	0.003 (3)	0.014 (4)	0.007 (3)
Eu1	0.01663 (10)	0.02304 (10)	0.01675 (10)	0.00095 (12)	0.00732 (7)	0.00014 (12)
N1	0.016 (2)	0.048 (3)	0.039 (3)	0.0046 (18)	0.0030 (19)	0.002 (2)
N2	0.021 (2)	0.052 (3)	0.035 (3)	−0.0045 (19)	0.0135 (19)	0.002 (2)
N3	0.022 (2)	0.064 (3)	0.031 (3)	0.003 (2)	0.0091 (18)	−0.009 (2)
N4	0.021 (2)	0.049 (3)	0.044 (3)	−0.0021 (19)	0.012 (2)	−0.007 (2)
N5	0.024 (2)	0.029 (2)	0.037 (3)	0.0042 (18)	0.005 (2)	0.0062 (18)
N6	0.026 (2)	0.022 (2)	0.040 (3)	−0.0045 (17)	0.009 (2)	−0.0010 (18)
O1	0.0202 (17)	0.0360 (19)	0.029 (2)	−0.0072 (14)	0.0032 (15)	−0.0032 (14)
O2	0.0261 (18)	0.043 (2)	0.034 (2)	−0.0138 (15)	0.0119 (16)	−0.0060 (16)
O3	0.0183 (17)	0.0327 (18)	0.0256 (18)	0.0060 (13)	0.0063 (14)	0.0054 (14)
O4	0.0321 (19)	0.069 (3)	0.026 (2)	0.0199 (18)	0.0120 (16)	0.0046 (17)
O5	0.0285 (17)	0.0204 (16)	0.0230 (17)	−0.0002 (13)	0.0080 (14)	−0.0008 (13)
O6	0.0311 (18)	0.0283 (17)	0.0244 (19)	−0.0013 (14)	−0.0016 (15)	0.0049 (14)

### Geometric parameters (Å, °)

**Table:**

C1—O1	1.255 (5)	C16—H16	0.9300
C1—O2	1.261 (5)	C17—O6	1.267 (5)
C1—C2	1.511 (6)	C17—O5	1.283 (5)
C2—C7	1.363 (6)	C17—C18	1.477 (6)
C2—C3	1.409 (7)	C18—C19	1.381 (7)
C3—C4	1.385 (7)	C18—C23	1.420 (7)
C3—H3	0.9300	C19—C20	1.381 (8)
C4—C5	1.381 (7)	C19—H19	0.9300
C4—H4	0.9300	C20—N6	1.377 (9)
C5—N1	1.391 (6)	C20—C21	1.428 (13)
C5—C6	1.398 (6)	C21—N5	1.370 (6)
C6—C7	1.383 (6)	C21—C22	1.399 (7)
C6—N2	1.406 (6)	C22—C23	1.367 (6)
C7—H7	0.9300	C22—H22	0.9300
C8—N1	1.317 (7)	C23—H23	0.9300
C8—N2	1.327 (6)	C24—N5	1.316 (7)
C8—H8	0.9300	C24—N6	1.346 (7)
C9—O4	1.258 (5)	C24—H24	0.9300
C9—O3	1.275 (5)	Eu1—O1	2.300 (3)
C9—C10	1.491 (6)	Eu1—O2^i^	2.320 (3)
C10—C11	1.385 (7)	Eu1—O4	2.357 (3)
C10—C15	1.421 (6)	Eu1—O6	2.417 (3)
C11—C12	1.384 (6)	Eu1—O5^i^	2.429 (3)
C11—H11	0.9300	Eu1—O3^i^	2.441 (3)
C12—N3	1.384 (6)	Eu1—O3	2.601 (3)
C12—C13	1.410 (6)	Eu1—O5	2.610 (3)
C13—C14	1.380 (7)	N1—H1	0.8600
C13—N4	1.384 (6)	N3—H3A	0.8600
C14—C15	1.376 (7)	N5—H5	0.8600
C14—H14	0.9300	O2—Eu1^ii^	2.320 (3)
C15—H15	0.9300	O3—Eu1^ii^	2.441 (3)
C16—N3	1.322 (6)	O5—Eu1^ii^	2.429 (3)
C16—N4	1.332 (7)		
			
O1—C1—O2	124.2 (4)	N5—C24—H24	122.8
O1—C1—C2	117.0 (4)	N6—C24—H24	122.8
O2—C1—C2	118.8 (4)	O1—Eu1—O2^i^	83.94 (12)
C7—C2—C3	121.2 (4)	O1—Eu1—O4	107.47 (13)
C7—C2—C1	119.7 (4)	O2^i^—Eu1—O4	160.37 (12)
C3—C2—C1	119.0 (4)	O1—Eu1—O6	129.67 (12)
C4—C3—C2	121.0 (4)	O2^i^—Eu1—O6	87.25 (11)
C4—C3—H3	119.5	O4—Eu1—O6	96.84 (13)
C2—C3—H3	119.5	O1—Eu1—O5^i^	80.62 (11)
C5—C4—C3	117.7 (5)	O2^i^—Eu1—O5^i^	79.58 (11)
C5—C4—H4	121.1	O4—Eu1—O5^i^	86.46 (12)
C3—C4—H4	121.1	O6—Eu1—O5^i^	145.61 (11)
C4—C5—N1	132.4 (4)	O1—Eu1—O3^i^	151.78 (11)
C4—C5—C6	120.5 (4)	O2^i^—Eu1—O3^i^	85.82 (12)
N1—C5—C6	107.1 (4)	O4—Eu1—O3^i^	76.66 (12)
C7—C6—C5	121.9 (4)	O6—Eu1—O3^i^	75.81 (11)
C7—C6—N2	130.3 (5)	O5^i^—Eu1—O3^i^	71.68 (10)
C5—C6—N2	107.8 (4)	O1—Eu1—O3	76.68 (11)
C2—C7—C6	117.6 (4)	O2^i^—Eu1—O3	147.53 (12)
C2—C7—H7	121.2	O4—Eu1—O3	52.10 (11)
C6—C7—H7	121.2	O6—Eu1—O3	85.47 (11)
N1—C8—N2	115.6 (5)	O5^i^—Eu1—O3	121.64 (11)
N1—C8—H8	122.2	O3^i^—Eu1—O3	122.60 (13)
N2—C8—H8	122.2	O1—Eu1—O5	78.07 (11)
O4—C9—O3	119.5 (4)	O2^i^—Eu1—O5	84.43 (11)
O4—C9—C10	119.1 (4)	O4—Eu1—O5	113.16 (11)
O3—C9—C10	121.4 (4)	O6—Eu1—O5	51.75 (10)
C11—C10—C15	121.1 (4)	O5^i^—Eu1—O5	154.53 (14)
C11—C10—C9	121.5 (4)	O3^i^—Eu1—O5	126.96 (10)
C15—C10—C9	117.4 (4)	O3—Eu1—O5	66.35 (10)
C12—C11—C10	117.3 (4)	O1—Eu1—Eu1^i^	114.44 (9)
C12—C11—H11	121.3	O2^i^—Eu1—Eu1^i^	68.71 (9)
C10—C11—H11	121.3	O4—Eu1—Eu1^i^	91.82 (9)
C11—C12—N3	131.6 (4)	O6—Eu1—Eu1^i^	107.90 (8)
C11—C12—C13	121.4 (4)	O5^i^—Eu1—Eu1^i^	37.71 (7)
N3—C12—C13	107.0 (4)	O3^i^—Eu1—Eu1^i^	37.56 (7)
C14—C13—N4	131.1 (4)	O3—Eu1—Eu1^i^	143.34 (7)
C14—C13—C12	121.3 (4)	O5—Eu1—Eu1^i^	147.99 (6)
N4—C13—C12	107.6 (4)	O1—Eu1—Eu1^ii^	63.70 (9)
C15—C14—C13	117.8 (5)	O2^i^—Eu1—Eu1^ii^	112.81 (9)
C15—C14—H14	121.1	O4—Eu1—Eu1^ii^	86.75 (9)
C13—C14—H14	121.1	O6—Eu1—Eu1^ii^	74.80 (8)
C14—C15—C10	121.1 (4)	O5^i^—Eu1—Eu1^ii^	139.58 (7)
C14—C15—H15	119.5	O3^i^—Eu1—Eu1^ii^	144.06 (7)
C10—C15—H15	119.5	O3—Eu1—Eu1^ii^	34.90 (7)
N3—C16—N4	114.6 (5)	O5—Eu1—Eu1^ii^	34.69 (6)
N3—C16—H16	122.7	Eu1^i^—Eu1—Eu1^ii^	177.100 (10)
N4—C16—H16	122.7	C8—N1—C5	105.5 (4)
O6—C17—O5	119.3 (4)	C8—N1—H1	127.3
O6—C17—C18	119.4 (4)	C5—N1—H1	127.3
O5—C17—C18	121.2 (4)	C8—N2—C6	104.0 (4)
C19—C18—C23	120.6 (4)	C16—N3—C12	105.7 (4)
C19—C18—C17	120.4 (5)	C16—N3—H3A	127.2
C23—C18—C17	118.7 (4)	C12—N3—H3A	127.2
C20—C19—C18	118.6 (7)	C16—N4—C13	105.1 (4)
C20—C19—H19	120.7	C24—N5—C21	105.7 (4)
C18—C19—H19	120.7	C24—N5—H5	127.1
N6—C20—C19	133.0 (11)	C21—N5—H5	127.1
N6—C20—C21	106.4 (5)	C24—N6—C20	105.5 (6)
C19—C20—C21	120.4 (8)	C1—O1—Eu1	137.9 (3)
N5—C21—C22	131.8 (5)	C1—O2—Eu1^ii^	132.7 (3)
N5—C21—C20	107.9 (4)	C9—O3—Eu1^ii^	157.8 (3)
C22—C21—C20	120.4 (4)	C9—O3—Eu1	86.9 (3)
C23—C22—C21	118.2 (5)	Eu1^ii^—O3—Eu1	107.53 (11)
C23—C22—H22	120.9	C9—O4—Eu1	98.7 (3)
C21—C22—H22	120.9	C17—O5—Eu1^ii^	131.8 (3)
C22—C23—C18	121.4 (4)	C17—O5—Eu1	88.2 (2)
C22—C23—H23	119.3	Eu1^ii^—O5—Eu1	107.59 (11)
C18—C23—H23	119.3	C17—O6—Eu1	97.5 (3)
N5—C24—N6	114.5 (5)		

Symmetry codes: (i) *x*, −*y*, *z*+1/2; (ii) *x*, −*y*, *z*−1/2.

### Hydrogen-bond geometry (Å, °)

**Table:**

*D*—H···*A*	*D*—H	H···*A*	*D*···*A*	*D*—H···*A*
N3—H3A···N2^iii^	0.86	1.96	2.798 (6)	165.
N5—H5···N4^iv^	0.86	2.04	2.839 (6)	155.
N1—H1···N6^iv^	0.86	1.97	2.827 (6)	173.

Symmetry codes: (iii) *x*+1/2, −*y*−1/2, *z*−1/2; (iv) *x*−1/2, −*y*+1/2, *z*−1/2.
